# Factors affecting hair cortisol concentration in privately owned intact cats

**DOI:** 10.3389/fvets.2026.1814440

**Published:** 2026-04-14

**Authors:** Veronika Vojtkovská, Naďa Konečná, Karla Štěpánková, Monika Šebánková, Simona Kovaříková

**Affiliations:** Department of Animal Protection and Welfare and Veterinary Public Health, Faculty of Veterinary Hygiene and Ecology, University of Veterinary Sciences Brno, Brno, Czechia

**Keywords:** animal welfare, caregiver, cortisol, hair, lifestyle

## Abstract

Understanding the factors affecting long-term stress in domestic cats is essential for welfare assessment, and hair cortisol concentration has been proposed as a promising biomarker for this purpose. This study investigated hair cortisol concentrations in intact, privately owned cats (*Felis silvestris catus*), with emphasis on physical and behavioral factors, as well as housing conditions. Hair samples were collected from 103 cats (40 males, 63 females) during routine preparation of the surgical field under general anesthesia prior to neutering. In females, samples were collected from the abdominal region along the *linea alba*, whereas in males, hair was taken from the area surrounding the scrotum. Information about the cats was collected from their owners, who completed a brief questionnaire covering the cats’ basic characteristics and lifestyle. Cortisol extraction from hair and subsequent quantification were performed using a commercially available ELISA kit. The overall mean hair cortisol concentration detected in the 103 cats was 9.99 pg./mg. Age, breed, coat type, coat color, and season of hair collection had no significant effect on cats’ hair cortisol levels (*p* > 0.05). However, sex was found to significantly influence hair cortisol concentration, with male cats exhibiting higher cortisol levels. Environmental and behavioral factors in cats were not found to significantly affect hair cortisol concentrations. These findings suggest that long-term cortisol levels in intact, privately owned cats may be influenced more by inherent individual traits than by environmental or behavioral factors, indicating a need for further research in this area.

## 1 Introduction

In mammals, cortisol detection is widely recognized as a biological indicator of stress response, reflecting activation of the hypothalamic–pituitary–adrenal (HPA) axis (1). With increasing emphasis on non-invasive welfare assessment, biological samples that can be collected without direct interference with the animal are preferred. Among these, hair provides a retrospective measure of cortisol accumulation over an extended period (1, 2). Hair cortisol concentration is not influenced by circadian rhythms or short-term physiological fluctuations, making it a useful biomarker for chronic stress (3). Chronic stress has a substantial impact on animal welfare and therefore warrants close attention. In cats, persistent stress has been shown to compromise immune barriers (4) and to diminish overall stress-coping capacity.

Cortisol incorporation into hair is thought to occur through three principal mechanisms: passive diffusion from blood capillaries surrounding the follicle during keratinization in the growth phase (5); diffusion from sweat or sebum during hair shaft formation (6); and extra-adrenal steroidogenesis by melanocytes within the skin and follicle (7). Cortisol may also reach the hair externally, for example, through grooming, as feline saliva contains measurable cortisol (1). However, it remains uncertain whether externally deposited cortisol is truly integrated into the hair shaft (8).

Hair sampling offers several advantages, including simple and non-invasive collection (9) and convenient long-term storage. Owing to its biochemical stability, hair can be stored at room temperature for months or even years without measurable alteration in cortisol content (10). Sampling is commonly performed using the ‘shave–reshave’ technique, in which a defined area is shaved at the start of the observation period and re-shaved after regrowth (11).

Multiple factors are believed to influence hair cortisol concentration in cats. Reported variables include age (9), coat color (12), sex (13), sampling site and method (14, 15), season (16), health status (12, 15, 17), neutering status (18), housing conditions (15, 19), and particular behavior (14, 19). Information on intact, privately owned companion cats remains limited, particularly regarding lifestyle-related factors. This study therefore, complements existing research by providing a more comprehensive assessment of multiple variables, with emphasis on those associated with the cats’ living conditions.

## 2 Materials and methods

### 2.1 Sample collection

Hair samples were obtained in collaboration with private veterinary practices providing small-animal care. Sampling was performed by a veterinary technician using electric clippers, during routine preparation of the surgical field under general anesthesia prior to neutering. All cats were intact at the time of hair sampling. In females, samples were collected from the abdominal region along the *linea alba*, whereas in males, hair was taken from the area surrounding the scrotum. The approximate sample size was 5 × 5 cm in females and 2 × 2 cm in males. In total, hair samples were collected from 103 cats (40 males, 63 females).

Owners were informed verbally and in writing about the scientific purpose of the study, and provided consent for the use of hair samples and accompanying data by completing a short questionnaire. The questionnaire included items on the cat’s basic characteristics, husbandry conditions, lifestyle, and behavior (see Supplementary material). Questions were selected based on previous studies addressing detecting the hair cortisol level in association with feline welfare and human–cat relationships (15, 19).

Immediately after collection, hair samples were placed in clean, resealable ZIP bags, labelled with identification codes and other relevant information, and transported together with completed owners’ questionnaires to the laboratory of the Department of Animal Protection and Welfare and Public Veterinary Health at the University of Veterinary Sciences Brno in the Czech Republic. Samples were stored at room temperature, protected from sunlight, until analysis.

### 2.2 Cortisol extraction and analysis

Cortisol extraction from hair and subsequent quantification were performed using a commercially available Cortisol Express ELISA kit (Cayman Chemical, USA; detection range 39.1–5,000 pg./mL), as it was previously used in assessing hair cortisol concentrations in dogs (20). To ensure optimal cortisol recovery and assay performance, manufacturer’s instructions was followed. All measured concentrations fell within the assay detection range, and therefore, no further adjustments were required. For each extraction, 40 ± 5 mg of each hair sample was weighed and mixed with 1.5 mL of hexane, then incubated for 2 min. After incubation, hexane was removed, and any remaining solvent was evaporated under a nitrogen stream until the samples were completely dry.

The dried samples were frozen at −20 °C for 20 min and then pulverised using a homogeniser (TissueLyser II, Qiagen, The Netherlands) following a protocol of 10 cycles of 2 min at 30 rpm. The resulting hair powder was mixed with 1.8 mL of methanol, vortexed, and incubated in a shaker (Thermo-Shaker PST-100HL, Biostan, Latvia) for 15 h at 25 °C and 1,030 rpm. After incubation, the mixture was filtered using PTFE syringe filters (25 mm, 0.22 μm; Labicom s.r.o., Czech Republic) to separate methanol from solid residues. Methanol was then evaporated under a nitrogen stream at 37 °C. The resulting residue was reconstituted in 400 μL of ELISA buffer (provided by the manufacturer in the ELISA kit).

Prepared samples were analysed in triplicate according to the manufacturer’s ELISA protocol. Absorbance was measured at 405 nm using a microplate reader (Varioskan Flash, Thermo Fisher Scientific, Finland).

### 2.3 Statistical analysis

Data were analysed using Unistat 6.5 software (Unistat Ltd., UK). Data normality was assessed using the Kolmogorov–Smirnov test. For non-normally distributed data, differences between two categorical groups were evaluated using the Mann–Whitney *U*-test. For comparisons involving three or more categorical groups, the Kruskal–Wallis ANOVA was applied, followed by a non-parametric Tukey-type *post hoc* test for pairwise comparisons. To reduce the risk of type I error due to multiple testing, *p*-values were adjusted using the Holm–Bonferroni correction. Correlations were assessed using Spearman’s rank correlation coefficient. Statistical significance was set at *p* ≤ 0.05.

## 3 Results

### 3.1 Description of the study population

Hair samples were collected from 103 cats (63 females and 40 males), aged 3 months to 8 years, undergoing neutering under general anaesthesia. The majority of owners identified their cats as non-pedigree domestic shorthaired and longhaired cats (*n =* 82), while 17 showed breed characteristics (Maine Coo*n =* 10, British Shorthair = 4, Russian Blue = 1, Exotic Shorthair = 1, Siamese = 1). In the case of 4 cats, owners did not give breed related specifications. Sixty-one samples were collected during autumn and winter (September to February) and 34 during spring and summer (March to August). Eight owners did not answer the question regarding the date of surgery. The majority of cats enrolled in the study were between 6 and 12 months of age (*n =* 73). An additional 16 cats were aged 0–6 months, while 14 cats were between 1 and 8 years old.

At the time of sampling, 84 cats were clinically healthy and were not receiving any medications or supplements that might affect hair cortisol concentration. Ten cats had health issues either at the time of sampling (ovarian cyst = 1, deafness = 1, dermatophytosis = 1, otitis = 2, FeLV infectio*n =* 1, unspecified problems = 1, orthopaedic problem = 1, infestation of *Otodectes cynotis* = 1) or within the previous 3 months (urinary crystals = 1). Owners of 9 cats did not give any specific information about the health state of their animals (they choose ‘I do not know’ type of answer). Two cats were receiving medicinal products (ofloxacin eye drops, *n =* 1; unspecified antibiotic, *n =* 1), and three cats were receiving nutraceuticals (unspecified vitamin supplements, *n =* 1; VetoMune for immune support, *n =* 1; unspecified joint supplements, *n =* 1) at the time of the study. Health status and medication use were included as categorical variables in the statistical analysis to assess their potential influence on hair cortisol concentrations.

Most cats (*n =* 88) were fed a mixed diet of dry and wet food (commercial canned/pouch products or raw meat). Ten cats had other feeding regimens: dry food only (*n =* 2), wet food only (*n =* 1), or combinations of dry food with other components (raw chicken, table scraps, yoghurt, or dried sprats; *n =* 6). One cat was fed vegan dry food combined with meat-based canned food. For 5 cats, the owners selected the answer “I do not know” in the questionnaire.

Regarding origin, 33 cats were found by their owners, 40 cats originated from friends and family, 13 originated from breeders, five cats were born in the owners’ household, and five cats were adopted from shelters. In the case of 7 cats, owners did not specify their origin (they chose ‘I do not know’ type of answer).

The duration of ownership before sampling was ≤3 months in 9 cats, 3–6 months in 41 cats, 6–12 months in 45 cats, and 1–8 years in 8 cats.

### 3.2 Effect of sex, age, breed, coat type, coat colour, health status, and season of sample collection on hair cortisol levels in cats

The overall mean hair cortisol concentration detected in the 103 cats was 9.99 pg./mg (median 5.54 pg./mg; range 2.37–170.46 pg./mg). Hair cortisol concentrations in relation to the assessed factors are presented in Table 1. Age, breed, coat type, coat colour, and season of hair collection had no significant effect on cats’ hair cortisol levels (*p* > 0.05). However, sex was found to significantly influence hair cortisol concentration, with male cats exhibiting higher cortisol levels compared to females (*p* = 0.0121).

**Table 1:** Cats’ hair cortisol concentrations in relation to sex, age, breed, hair type, coat colour, health status, and season of hair sample collection.

Factor	Category	*N*	Mean (pg/mg)	Median (pg/mg)	*P*-value
Sex	Male	40	11.36	7.16	0.0121^a^
	Female	63	9.12	5.03	
Age	0–6 months	16	20.21	7.40	
	6 months–1 year	73	8.03	4.92	0.0529^b^
	1–8 years	14	8.50	6.98	
Breed	Domestic cat	82	10.62	5.79	0.1025^a^
	Other (breed-related)	17	8.12	4.40	
Hair type	Long and semi-long	22	14.40	6.15	0.9743^a^
	Short	81	8.80	5.22	
Coat color	Light (white, ginger, cream, or combination)	34	7.23	13.30	0.4761^b^
	Dark (black, brown, or combination)	27	5.11	6.10	
	Mixed (dark + light)	42	10.09	5.16	
Health state	Cats with health problems	10	27.86	8.42	0.8181^a^
	Healthy cats	84	7.08	5.60	
Sampling season	September–February	61	9.39	5.86	0.4655^a^
	March–August	34	11.27	4.98	

^a^Mann–Whitney *U*-test; ^b^Kruskal Wallis ANOVA.

### 3.3 Effect of way of life on hair cortisol levels in cats

Lifestyle factors in cats (strictly indoor, strictly outdoor, or a combination of both) were not found to significantly affect hair cortisol concentrations (*p* = 0.3328). Five breeders answered the question about the cats’ lifestyle with “I do not know” type of answer; therefore, these responses were not included in the analysis. Likewise, the presence of other animals or children in the household did not have a statistically significant impact. However, mean and median cortisol levels tended to be higher in cats cohabiting with other animals or children (Table 2).

**Table 2:** Hair cortisol levels in relation to cats’ lifestyle.

Factor	Category	*N*	Mean (pg/mg)	Median (pg/mg)	*P*-value
Way of life	Indoor	43	6.67	5.66	0.3328^b^
	Outdoor	27	13.53	6.09	
	Combination	28	8.06	5.12	
N + 1 rule for beds and hiding places was followed^†^	Yes	28	11.60	5.06	0.9882^a^
	No	32	6.53	4.69	
N + 1 rule for cat toilets was followed^†^	Yes	12	19.42	5.73	0.9451^a^
	No	52	7.36	5.12	
Presence of any other animals (cats or other species) in a household^†^	Yes	51	10.78	5.72	0.1635^a^
	No	20	5.94	4.73	
Presence of other cats in a household^†^	Yes, a cat is sharing a household with at least one other cat	40	8.12	6.32	0.1628^a^
	No, the cat is the only animal in the household	20	5.94	4.73	
Presence of a child/children in a household^†^	Yes	25	6.69	5.16	0.9424^a^
	No	46	10.89	5.23	

^a^Mann–Whitney *U*-test, b Kruskal Wallis ANOVA. ^†^Only cats living indoor or cats living both indoor and outdoor were involved in the analysis.

No significant association was detected between hair cortisol concentration and the duration of ownership (r_s_ = −0.0598; *p* = 0.5482). Feeding regime – specifically, feeding a combination of dry and wet food (*n =* 88) compared with other feeding types (*n =* 10) – did not affect hair cortisol concentration (*p* = 0.1053).

No significant correlations were also identified between the number of animals (of any species and other cats) and children sharing a household (or outdoor environment) with the study cats (Table 3). Similarly, no significant correlations were found between the number of resources provided within the household and hair cortisol concentration (Table 4).

**Table 3:** Correlation analysis of the number of other animals and children sharing the household with the studied cat population and the hair cortisol concentration.

Indicator	Hair cortisol concentration
Number of animals (any species) sharing a household with cats (all lifestyles)	r_s_ = 0.1654; *p* = 0.1074
Number of animals (any species) sharing a household with cats (living only indoors or with a combined indoor–outdoor lifestyle)	r_s_ = 0.1551; *p* = 0.1964
Number of cats sharing a household with study cats (all lifestyles)	r_s_ = −0.1219; *p* = 0.2393
Number of cats sharing a household with study cats (living only indoors or with a combined indoor–outdoor lifestyle)	r_s_ = −0.1075; *p* = 0.3759
Number of children sharing a household with cats (all lifestyles)	r_s_ = −0.0179; *p* = 0.8631
Number of children sharing a household with cats (living only indoors or with a combined indoor–outdoor lifestyle)	r_s_ = −0.0451; *p* = 0.7089

**Table 4:** Correlation analysis of the number of resources provided to cats in the household and the hair cortisol concentration.

Indicator	Hair cortisol concentration
Number of hiding places in a household (cats of all lifestyles included)	r_s_ = −0.01074; *p* = 0.3429
Number of hiding places in a household (cats living exclusively indoors or those with a combined indoor–outdoor lifestyle)	r_s_ = 0.0297; *p* = 0.8248
Number of litter trays in a household (cats living exclusively indoors or those with a combined indoor–outdoor lifestyle)	r_s_ = 0.0985; *p* = 0.4350
Number of scratching posts in a household (cats living exclusively indoors or those with a combined indoor–outdoor lifestyle)	r_s_ = 0.0744; *p* = 0.2795

### 3.4 Effect of behavior and household changes on hair cortisol concentration in cats

Behavioral factors and environmental changes were also analysed in relation to hair cortisol concentration. No significant association was observed between the level of sociability, as rated by owners on a 10-point scale (1 = extremely unsociable cat; e.g., timid, avoiding contact or consistently reacting aggressively, to 10 = highly socialized, affectionate or even demanding, strongly bonded to humans), and hair cortisol concentration (r_s_ = 0.1299; *p* = 0.2002).

Data on hair cortisol concentration in cats reported by owners to display undesirable behaviors, as well as in cats from households where owners indicated that one or more changes had occurred within the previous 3 months, are presented in Table 5.

**Table 5:** Hair cortisol levels in relation to undesirable behavior occurrence and changes in the household in the previous 3 months.

Factor	Category	*N*	Mean (pg/mg)	Median (pg/mg)	*P*-value
Undesirable behavior occurrence^†^	Yes	30	11.51	5.97	0.7982^a^
	No	70	9.56	5.20	
Changes in household in the previous 3 months ^‡^	Yes	23	9.26	6.68	0.1084^a^
	No	75	10.55	5.16	

^a^Mann–Whitney *U*-test. ^†^One or more forms of undesirable behavior: urine marking or urination/defecation outside the litter box, aggressive behavior without an apparent cause, destruction of household items, hiding/avoiding contact, and loss of appetite. ^‡^One or more environmental changes that had occurred in the household during the previous 3 months: relocation, renovation of the living or outdoor areas, the addition of a new family member (e.g., a child), the introduction of a new animal into the household.

## 4 Discussion

The present study aimed to analyse hair cortisol levels in relation to physical, environmental and behavioral factors in intact, privately owned companion cats. We found age, breed, coat type, coat color, and season of hair collection had no significant effect on cats’ hair cortisol levels (*p* > 0.05). However, sex was found to significantly influence hair cortisol concentration, with male cats exhibiting higher cortisol levels. Environmental and behavioral factors in cats were not found to significantly affect hair cortisol concentrations.

The mean hair cortisol level identified in this study was 9.99 pg./mg (median 5.54 pg./mg; range 2.37–170.46 pg./mg). Similar values were reported by Rothlin-Zachrisson et al. (15), who documented a median cortisol level of 4.8 pg./mg in abdominal hair. In their study, higher levels were observed when samples were collected from the forelimb (median 5.6 pg./mg) and when hair was obtained by combing from the back, lateral thorax and abdomen (median 7.2 pg./mg). Sampling location may therefore influence measured hair cortisol concentrations. This finding aligns with Contreras et al. (14), who reported that among various body regions (dorsal and ventral neck, lumbosacral area, abdomen), the highest cortisol concentrations were found on the ventral neck (median 17.7 pg./mg; range 3–224.1 pg./mg), whereas the lowest were recorded on the abdomen (median 5.5 pg./mg; range 2.7–10.8 pg./mg). One explanation for these differences may be the variable proportion of hair follicles in anagen, catagen and telogen phases, as well as natural shedding rhythms. Cortisol is incorporated into hair primarily during the anagen phase, and body areas with a higher proportion of follicles in anagen may therefore yield higher concentrations (21). Additional factors that may contribute to variation include contamination (e.g., with faeces), differential exposure of body regions to environmental conditions, grooming behavior, and variation in hair growth rate and skin perfusion (22, 23, 24, 25). Our results show no effect of breed, hair type or hair colour on hair cortisol concentrations in cats. Sex, however, had a significant influence, with males exhibiting higher hair cortisol concentrations. It remains unclear to what extent this difference may be explained by sampling site, as in our study hair was collected from different anatomical regions in males and females prior to neutering. It is also questionable to what extent the area from which hair was collected in male cats may have been contaminated with urine (or possibly saliva), and thus exposed to an external source of cortisol.

Regarding differences in hair cortisol concentrations in relation to neutering status, in the study by Rothlin-Zachrisson et al. (15), the majority of cats were neutered. The median cortisol concentration reported in their study was similar to that observed in the present study, although only intact individuals were included in our sample. Previous findings have suggested that higher hair cortisol concentrations in cats may be associated with neutering status, with higher values reported in intact individuals (18). However, that study included only female cats, and the sampling site varied depending on the cats’ temperament; approximately 30% of cats were sampled under general anesthesia, while the remaining individuals were sampled from the sacral area. Lower median hair cortisol concentrations in three intact and 31 neutered cats (2.29 pg./mg) were reported by Lima et al. (26), where samples were collected from the dorsal region. It remains unclear which factors contributed to the lower hair cortisol concentrations observed in that study and whether neutering status played a role. Nevertheless, to our knowledge, the present study is the first to investigate hair cortisol concentrations in a relatively large sample consisting exclusively of intact cats.

Although several studies have reported no sex-related differences in hair cortisol (14, 17, 19, 27, 28), Wojtaś et al. (29) documented sex differences in shelter cats. In their study, females had higher cortisol levels prior to shelter admission, whereas males showed higher levels after 4–5 weeks in the shelter environment. The authors did not provide an explanation for this pattern. Rothlin-Zachrisson et al. (15) reported that in clipped front leg hair, females with reported experiences of pregnancy or estrus had higher hair cortisol than spayed females. In combed hair, intact males had higher HCC than spayed females. Relevant factors that may underlie sex differences include social status, territorial behavior, body condition, and the influence of sex hormones on HPA-axis activity (30, 31, 32, 33).

Although an effect of coat colour on cortisol concentration has been described in domestic dogs (*Canis familiaris*) (12, 34), no such effect was identified in our study or in the work of Contreras et al. (14). In contrast, Nutter et al. (28) reported significantly higher cortisol concentrations in black hair compared with white hair. Ginger hair also tended to show higher levels, although the difference was not statistically significant. The protein responsible for coat pigmentation interacts with the melanocortin pathway, which influences cortisol production (35). However, the mechanisms governing cortisol incorporation into hairs of different colours remain unclear and require further investigation (10). Seasonal variation may also influence hair cortisol concentrations. Studies in dogs have reported higher levels during winter (3, 16). In our study, no statistically significant seasonal differences were found.

Analysis of age-related effects revealed that the highest cortisol concentrations occurred in the youngest age category (cats ≤ 6 months), with values that were close to, but not statistically significant. Galuppi et al. (17) reported no significant influence of age on hair cortisol in cats. However, in this study, only 14 cats (out of a total of 245) were younger than 1 year, and it was not specified how many of them were under 6 months of age. Heimbürge et al. (9) found elevated cortisol concentrations in calves compared with 6-month-old, 18-month-old, and adult cows (*Bos taurus*). Higher cortisol levels in young animals may be associated with lower concentrations of corticosteroid-binding globulin, resulting in a greater proportion of free cortisol in circulation, which is subsequently incorporated into hair at higher levels (9).

In the study by Rothlin-Zachrisson et al. (15), the presence of chronic disease was associated with higher hair cortisol concentrations. Comparison of hair cortisol levels in clinically healthy and hospitalized dogs was also conducted by Galdhar et al. (36), who reported significantly higher levels in hospitalized animals. Elevated cortisol in animals with diseases likely reflects physiological processes related to illness (37), as well as associated stressors such as veterinary visits, transport (38), and medication administration. Although higher cortisol levels were also observed in cats with health issues in our study, the difference between healthy and unhealthy cats was not statistically significant, likely due to the low number of diseased individuals and the predominantly mild nature of their medical conditions.

Consistent with the findings of Contreras et al. (14) and Nutter et al. (28), cats with outdoor access –or those living exclusively outdoors – did not exhibit lower cortisol levels than indoor-only cats in our study. Similarly, Wojtaś (19) found no significant differences in hair cortisol collected from the lumbosacral region of 55 companion cats, although a trend toward lower cortisol was observed in cats with outdoor access. This may reflect challenges associated with adapting to a strictly indoor lifestyle. Domestic cats were not selectively bred to function as typical companion animals or to live in confined indoor spaces in close proximity to humans (39). Indoor environments often lack opportunities for natural behaviors, and inadequate environmental management or insufficient resource provision may contribute to chronic stress, ultimately affecting cortisol levels.

For this reason, our study also examined the availability of key resources within the home environment. The number of resources – hiding places, litterboxes, and scratching posts – did not significantly correlate with hair cortisol levels. Moreover, compliance with the informal n + 1 rule for hiding places and litterboxes was not associated with cortisol concentrations. However, the finding that only 19% of indoor cats or cats with combined indoor-outdoor lifestyles lived in households that met the n + 1 guideline for litterboxes highlights substantial deficiencies in resource provision. This is particularly concerning in multi-cat households, where resources are shared among individuals.

Although our study did not identify statistically significant differences in hair cortisol concentrations between cats displaying owner-reported undesirable behaviors and those without such behaviors, a tendency toward higher cortisol levels was observed in cats exhibiting undesirable behaviors. This aligns with findings from Wojtaś (19), who reported higher hair cortisol in cats eliminating outside the litterbox or showing aggression toward household members, as well as results from Nutter et al. (28), who documented elevated cortisol in cats consuming non-food items, attacking harder during play, displaying excessive vocalization or scratching furniture. Undesirable behaviors often arise from unmet or misunderstood feline needs, which may compromise welfare and contribute to chronic stress.

Similar to our findings, neither Wojtaś (19) nor Contreras et al. (14) observed a relationship between hair cortisol levels and the number of cats living in the household. In contrast, Rothlin-Zachrisson et al. (15) reported higher cortisol levels in cats living in multi-cat households. Although no statistically significant differences were detected in our study, a trend toward lower cortisol concentrations was observed in cats living as the only pet in the household. Such cats may not face the social stress associated with sharing space and resources with other cats. In multi-cat households, resource sharing can lead to competition and aggression, which in turn may contribute to stress-related behaviors (40).

Exposure to unpredictable child behavior and household noise has been associated with increased stress in cats, manifested through signs such as decreased appetite, vomiting, and diarrhoea (41). In our study, however, no differences in hair cortisol levels were observed between cats living with children and those in child-free households. Nevertheless, Hart et al. (42) noted that child–cat relationships may be perceived as more problematic than adult–cat relationships. Relationships between a single cat and a child were described as less friendly compared with households where multiple cats were present. Cats living as the only pet may receive more focused attention and may struggle to avoid excessive interaction with children – potentially contributing to increased stress (43).

Our study also did not confirm an association between willingness to interact with the owner and hair cortisol concentrations. Most owners rated their cats’ willingness to interact as high (mean score 8.3). Future research could benefit from assessing cortisol levels across cats with formally defined personality profiles, as owner-based assessments of sociability may be highly subjective and reflect the owner–cat relationship rather than intrinsic personality traits. Studies using faecal cortisol metabolites found no correlation with personality dimensions such as agreeableness, openness and extraversion (44). In regularly fed, free-roaming urban cat groups, Finkler and Terkel (45) reported a positive correlation between hair cortisol levels and dominance scores in intact females, suggesting that dominance status may influence stress responses.

## 5 Conclusion

Chronic stress can substantially affect the overall welfare of companion cats. As many owners are unable to objectively assess their cats’ quality of life – whether due to limited knowledge of feline needs and behavior, anthropomorphism, unrealistic expectations, or a lack of emotional attachment that would otherwise motivate greater attention – quantification of hair cortisol offers a promising and easily applicable tool for objective welfare assessment.

The findings of this study should be interpreted in light of several limitations, including the relatively small sample size, the inherent subjectivity of owner-reported assessments in the questionnaire, uneven group sizes arising from characteristics of cats presented for neutering, slight differences in sampling locations between males and females, and the possibility of external cortisol contamination due to chosen sampling sites. Moreover, several factors influencing hair cortisol concentrations remain insufficiently understood. Future studies should consider examining hair cortisol in relation to individual temperament profiles, as wide inter-individual variability in stress responses is likely linked to underlying personality traits.

## Data Availability

The raw data supporting the conclusions of this article will be made available by the authors, without undue reservation.
