# Supplementary figures and images for: Factors affecting hair cortisol concentration in privately owned intact cats
Source: Front Vet Sci. 2026 Apr 14;13:1814440. doi: 10.3389/fvets.2026.1814440 (PMC13120955; doi:10.3389/fvets.2026.1814440)

Supplementary Material


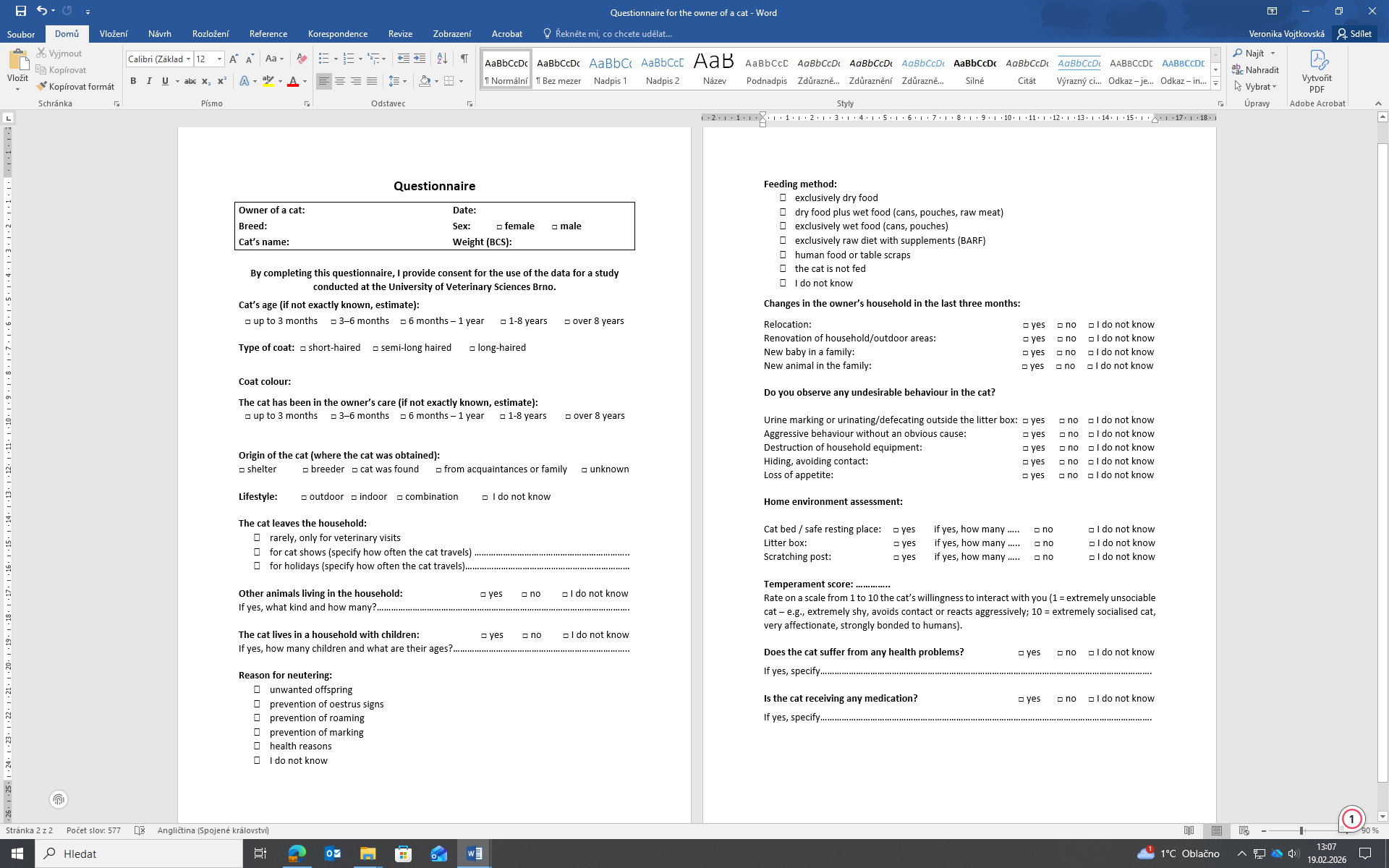


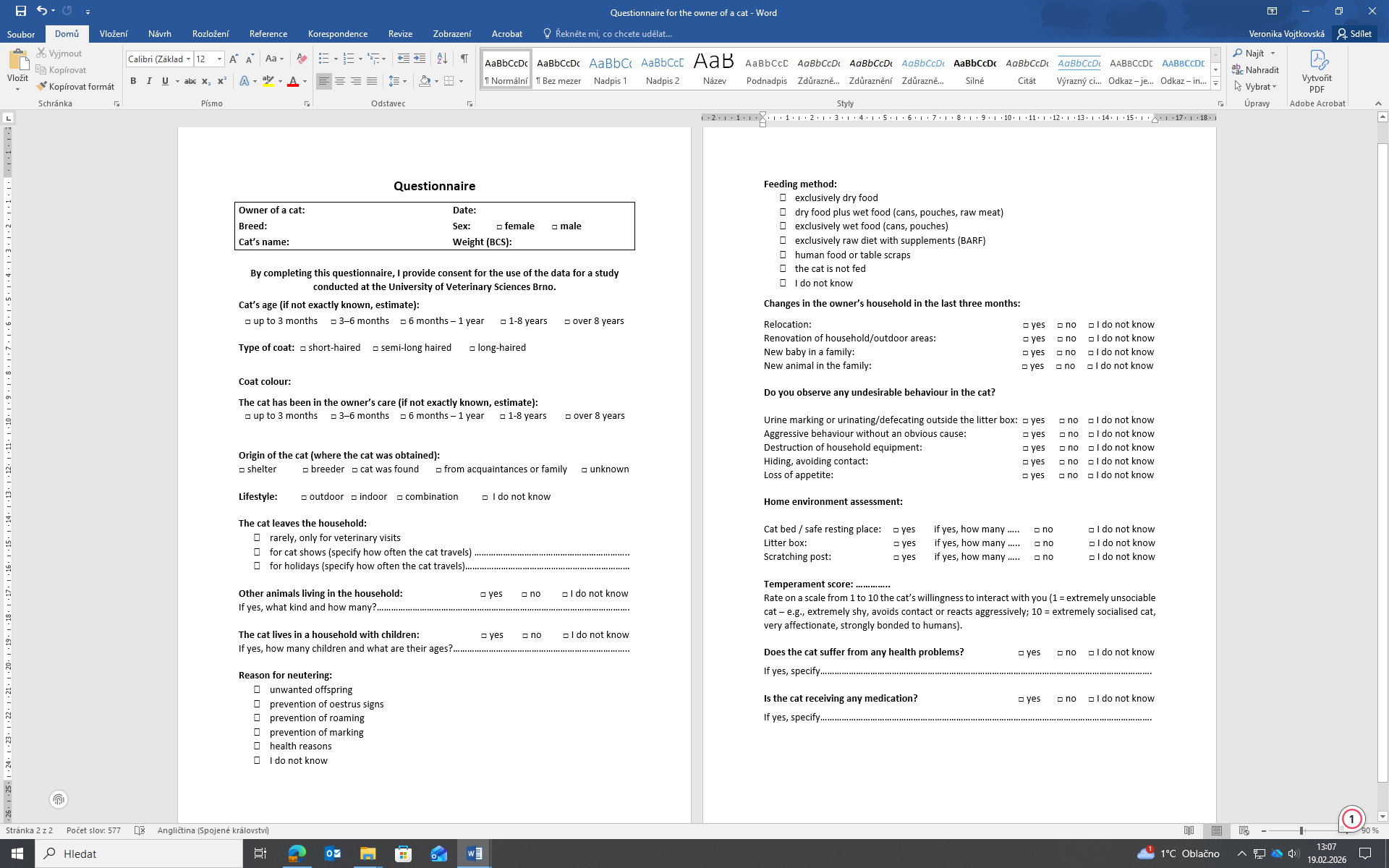

Supplement: Supplementary file 1 [file Table_1.DOCX]
